# Cost-effectiveness of tumor-treating fields plus standard therapy for advanced non-small cell lung cancer progressed after platinum-based therapy in the United States

**DOI:** 10.3389/fphar.2024.1333128

**Published:** 2024-02-05

**Authors:** Wentao Tian, Jiaoyang Ning, Liu Chen, Yu Zeng, Yin Shi, Gang Xiao, Shuangshuang He, Guilong Tanzhu, Rongrong Zhou

**Affiliations:** ^1^ Department of Oncology, Xiangya Hospital, Central South University, Changsha, China; ^2^ Changsha Stomatological Hospital, Hunan University of Traditional Chinese Medicine, Changsha, China; ^3^ Department of Pharmacy, Xiangya Hospital, Changsha, China; ^4^ Department of Radiation Oncology and Department of Head and Neck Oncology, Cancer Center, West China Hospital, Sichuan University, Chengdu, Sichuan, China; ^5^ Xiangya Lung Cancer Center, Xiangya Hospital, Central South University, Changsha, China; ^6^ National Clinical Research Center for Geriatric Disorders, Xiangya Hospital, Central South University, Changsha, Hunan, China

**Keywords:** advanced non-small cell lung cancer, tumor treating fields, immunotherapy, chemotherapy, cost-effectiveness

## Abstract

**Background:** Tumor treating fields (TTF) was first approved for treatment of glioblastoma. Recently, the LUNAR study demonstrated that TTF + standard therapy (ST) extended survival in patients with advanced non-small cell lung cancer (NSCLC). This primary objective of this study is to analyze the cost-effectiveness of this treatment from the United States healthcare payers’ perspective.

**Methods:** A 3-health-state Markov model was established to compare the cost-effectiveness of TTF + ST and that of ST alone. Clinical data were extracted from the LUNAR study, supplemented by additional cost and utility data obtained from publications or online sources. One-way sensitivity analysis, probabilistic sensitivity analysis, and scenario analysis were conducted. The willingness-to-pay (WTP) threshold per quality-adjusted life-years (QALYs) gained was set to $150,000. The main results include total costs, QALYs, incremental cost-effectiveness ratio (ICER) and incremental net monetary benefit (INMB). Subgroup analyses were conducted for two types of ST, including immune checkpoint inhibitor, and docetaxel.

**Results:** During a 10-year time horizon, the costs of TTF + ST and ST alone were $431,207.0 and $128,125.9, and the QALYs were 1.809 and 1.124, respectively. The ICER of TTF + ST compared to ST was $442,732.7 per QALY, and the INMB was -$200,395.7 at the WTP threshold. The cost of TTF per month was the most influential factor in cost-effectiveness, and TTF + ST had a 0% probability of being cost-effective at the WTP threshold compared with ST alone.

**Conclusion:** TTF + ST is not a cost-effective treatment for advanced NSCLC patients who progressed after platinum-based therapy from the perspective of the United States healthcare payers.

## Introduction

Non-small cell lung cancer (NSCLC) stands as the leading cause of cancer-related death, with a 5-year survival rate of approximately 23% (Siegel et al., 2023). Despite advancement in imaging technology and the widespread adoption of CT screening, over 55% of patients are still diagnosed at an advanced stage (Siegel et al., 2020). For individuals with metastatic NSCLC, the prospect of surgical cure diminishes. Systemic treatments such as targeted therapy, immunotherapy, radiotherapy, and chemotherapy serve to prolong their survival and improve the quality of life, (Chen et al., 2020). The predominant approach for patients with driver gene-negative advanced NSCLC is platinum-based chemotherapy (Clegg et al., 2001), but the inevitability of subsequent resistance poses a substantial challenge (Bluthgen and Besse, 2015; Barnfield and Ellis, 2016). Thus, finding new treatments is an urgent problem that needs to be solved.

Tumor treating fields (TTF) is a non-invasive tumor physical therapy, demonstrating significant therapeutic efficacy, convenience, and minimal adverse reactions in recurrent glioblastoma (Kirson et al., 2004). Further, the National Comprehensive Cancer Network (NCCN) recommended TTF for treating recurrent and newly diagnosed glioblastoma in 2013 and 2016, respectively (Petoukhova et al., 2023; Swartz et al., 2023). Preclinical research focusing on NSCLC suggests that TTF induces immunogenic death of tumor cell, enhances antigen presentation of dendritic cells and leukocyte chemotaxis, and synergizes with PD-1/PD-L1 inhibitors to inhibit tumor growth (Giladi et al., 2014; Giladi et al., 2015; Karanam et al., 2017; Shteingauz et al., 2018; Voloshin et al., 2020). Meanwhile, the combination of TTF with chemotherapy agents, such as cisplatin, paclitaxel, and pemetrexed has demonstrated inhibitory effects on NSCLC growth (Giladi et al., 2014). In terms of clinical applications for NSCLC patients, the phase 1/2 EF-15 (NCT00749346) clinical trial revealed that second-line TTF, in combination with chemotherapy, extended the median overall survival (mOS) of 42 patients with stage IIIB (with pleural effusion) or IV NSCLC to 13.8 months (Pless et al., 2013). Recently, the LUNAR (NCT02973789) trial confirmed the favorable efficacy and safety of TTF in combination with standard therapy (ST), including immune checkpoint inhibitor (ICI) or docetaxel, for metastatic NSCLC that has progressed after platinum-based therapy.

The LUNAR trial was a randomized, open-label, pivotal phase 3 study that included 276 patients from 130 regions in 19 countries (Leal et al., 2023). The mOS of the TTF + ST group and that of the ST group were 13.2 months and 9.5 months, respectively. Subgroup analysis indicated that the mOS of the TTF + docetaxel group was 11.1 month, while that of the docetaxel alone group was 8.7 months. Similarly, the TTF + ICI group exhibited a mOS of 18.5 months, whereas the ICI group had a mOS of 10.8 months. The median progression-free survival (mPFS) was 8 months and 4.1 months respectively in the TTF + ST group and the ST group. The mPFS was 4.4 months in the TTF + docetaxel group and 4.2 months in the docetaxel alone group. Similarly, the TTF + ICI group displayed a mPFS of 5.9 months, compared to 4.0 months in the ICI alone group (Leal et al., 2023).

Here, we aimed to investigate the cost-effectiveness of TTF + ST in the treatment of advanced NSCLC which had progressed after platinum-based chemotherapy, and explore the cost-effectiveness within subgroups by ST type, including ICI and docetaxel.

## Materials and methods

This study strictly follows the Consolidated Health Economic Evaluation Reporting Standards 2022 (CHEERS 2022). We used R (version 4.2.1) for analyzation and visualization.

### Model construction

**FIGURE 1:**
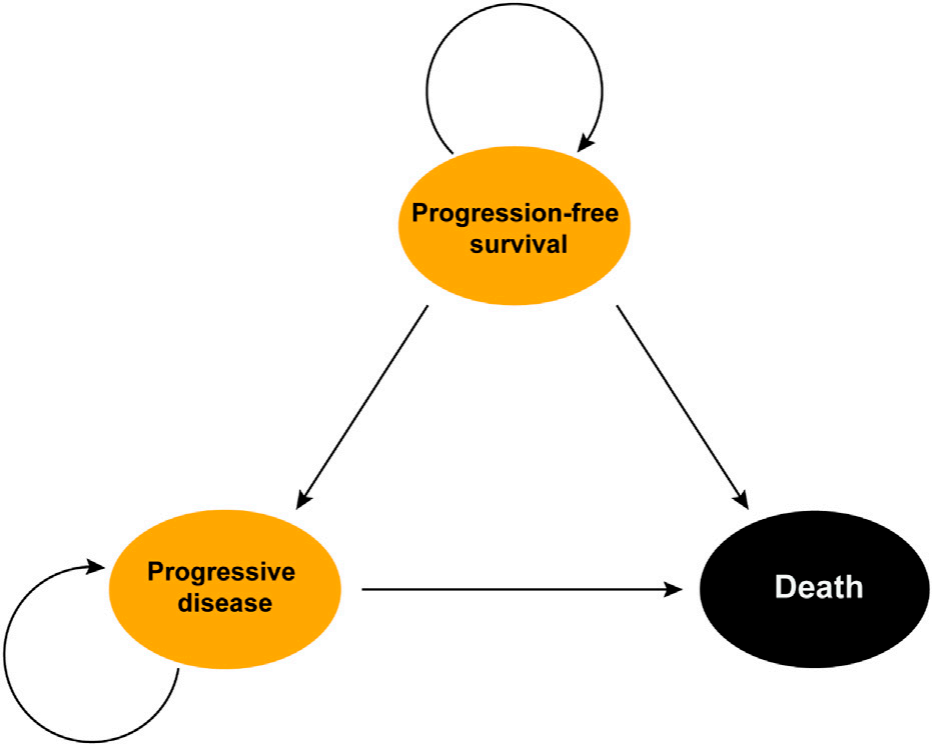
Structure of the Markov model. Death represents the state of absorption.

We utilized the “heemod” package to construct a Markov model featuring 21-day cycles for simulating the health status transitions of the patients in the LUNAR study. The model included three states, PFS, progressive disease (PD), and death, with death as the absorbing state (Kevin Zarca et al., 2023; Leal et al., 2023). The time horizon was set to 10 years, considering that over 95% of patients were assumed to be dead after 10 years of treatment. The transition probabilities from PFS to PD and from PD to death was determined based on the PFS and OS data and/or natural modality rate. Additionally, the transition probability from PFS to death was estimated to be equal to the natural modality rate automatically obtained from the “heemod” package (Kevin Zarca et al., 2023) (Figure 1).

In the LUNAR study, 137 patients underwent TTF (150 kHz, 18 h per day, NovoTTF device system) + ST, while 139 patients received ST [ICIs: nivolumab, pembrolizumab, or atezolizumab; or docetaxel]. Among patients who received ICIs, 2/3 were treated with nivolumab, 1/6 with pembrolizumab, and 1/6 with atezolizumab (Leal et al., 2023). To resemble the design of the LUNAR study, we defined the docetaxel treatment as receiving 75 mg/m^2^ of docetaxel over 1 h every 3 weeks, the ICI treatment as taking pembrolizumab (200 mg every 3 weeks), atezolizumab (1,200 mg every 3 weeks), or nivolumab (240 mg every 2 weeks) in a 1:1:2 ratio. The TTF treatment was defined as receiving TTF every day. Furthermore, the TTF + ST was defined as undergoing TTF + ICI or TTF + docetaxel in a 66:71 ratio, and the ST was defined as taking ICI or docetaxel alone in a 71:68 ratio (Leal et al., 2023).

We set a relatively high willingness-to-pay (WTP) threshold of $150,000 per quality-adjusted life-years (QALYs) gained because of the high inflation rate in the United States in recent years and an increasing popularity of this WTP threshold in cost-effectiveness analyses nowadays (Neumann et al., 2014; Neumann and Kim, 2023). As for cost-effectiveness results, we focused on overall costs, QALYs, ICERs, and incremental net monetary benefit (INMB) to evaluate cost-effectiveness.

### Clinical data

OS and PFS data for the TTF + ST, ST, TTF + ICI, IC, TTF + docetaxel, and docetaxel groups were extracted from the LUNAR study using the GetData Graph Digitizer (version 2.26). Subsequently, the “IPDfromKM” package (Liu et al., 2021) was employed to reconstruct individual patient data, which were then adopted to recreate Kaplan-Meier Curves to ensure the accuracy (Supplementary Figure S1).

To obtain long-term OS and PFS survival curves, we applied a variety of distributions in the “flexsurv” package (including exponential, Weibull, log-logistic, log-normal, generalized gamma, gamma, Gompertz, and Roystone/Parmar distributions) to fit the individual patient data. The Akaike Information Criterion (AIC) and Bayesian Information Criterion (BIC) values were calculated to identify the best distribution for each dataset (Supplementary Table S1). Additionally, we collected the incidences of various adverse events, discontinuation rates, and subsequent treatments after progression for the main analysis (Table 1) and subgroup analyses (Supplementary Table S2), from the LUNAR study.

**TABLE 1:** Parameters input to the model.

Parameters	Base value	Range		Distribution	Source
		Minimum	Maximum		
Clinical					
Body weight	70	52.5	87.5	Normal	Liu et al. (2021b)
BSA	1.8	1.35	2.25	Normal	Liu et al. (2021b)
Discount rate	0.05	0	0.08	Uniform	Smith and Gravelle (2001)
Treatment Discontinuation rate in the TTF + ST group	0.35	0.26	0.44	Beta	Leal et al. (2023)
Treatment Discontinuation rate in the ST group	0.19	0.15	0.24	Beta	Leal et al. (2023)
Royston-Parmar OS survival model of the TTF + ST	Gamma0: −3.444	ND	ND	ND	Model fitting
	Gamma1: 0.528				
	Gamma2: −0.349				
	Gamma3: 0.483				
Exponential model for OS of ST	Rate: 0.068	ND	ND	ND	Model fitting
Generalized gamma model for PFS of TTF + ST	Mu: 1.24	ND	ND	ND	Model fitting
	Sigma: 1.035				
	Q: −0.728				
Generalized gamma model for PFS of ST	Mu: 1.037	ND	ND	ND	Model fitting
	Sigma: 0.964				
	Q: −0.872				
Treatment cost, $					
TTF (per month)	27,214.48	20,410.86	34,018.1	Gamma	Bernard-Arnoux et al. (2016)
Pembrolizumab (per mg)	54.81	41.11	68.51	Gamma	De Marzi et al. (2020)
Nivolumab (per mg)	30.46	22.85	38.08	Gamma	De Marzi et al. (2020)
Atezolizumab (per mg)	8.27	6.2	10.34	Gamma	De Marzi et al. (2020)
Docetaxel (per mg)	0.94	0.71	1.18	Gamma	De Marzi et al. (2020)
Other costs, $					
Drug Administration	132.16	99.12	165.2	Gamma	Dutt et al. (2020)
Laboratory tests and scans per cycle	268.00	201.00	335.00	Gamma	Zhu et al. (2023)
Hospitalization per day	76.62	57.47	95.78	Gamma	De Marzi et al. (2020)
Best supportive care per cycle	2,603.31	1,952.49	3,254.14	Gamma	Criss et al. (2019)
End of life	17,909.24	13,431.93	22,386.55	Gamma	Dutt et al. (2020)
Follow up per cycle	36.86	27.65	46.08	Gamma	Klein et al. (2010)
Subsequent therapy cost, $					
Bevacizumab (per mg)	7.36	5.52	9.2	Gamma	De Marzi et al. (2020)
Carboplatin (per mg)	0.05	0.04	0.06	Gamma	De Marzi et al. (2020)
Cisplatin (per mg)	0.32	0.24	0.4	Gamma	De Marzi et al. (2020)
Crizotinib (per mg)	1.49	1.12	1.86	Gamma	De Marzi et al. (2020)
Erlotinib (per mg)	1.70	1.28	2.13	Gamma	De Marzi et al. (2020)
Etoposide (per mg)	0.70	0.53	0.88	Gamma	De Marzi et al. (2020)
Gemcitabine (per mg)	0.02	0.02	0.03	Gamma	De Marzi et al. (2020)
Nab_paclitaxel (per mg)	12.06	9.05	15.08	Gamma	De Marzi et al. (2020)
Paclitaxel (per mg)	0.12	0.09	0.15	Gamma	De Marzi et al. (2020)
Pemetrexed (per mg)	0.99	0.74	1.24	Gamma	De Marzi et al. (2020)
Vinorelbine (per mg)	0.83	0.62	1.04	Gamma	De Marzi et al. (2020)
Subsequent therapy proportion in the TTF + ST group					
Bevacizumab	0.00	0.00	0.01	Beta	Leal et al. (2023)
Carboplatin	0.17	0.13	0.21	Beta	Leal et al. (2023)
Cisplatin	0.02	0.02	0.03	Beta	Leal et al. (2023)
Crizotinib	0.00	0.00	0.01	Beta	Leal et al. (2023)
Erlotinib	0.37	0.28	0.46	Beta	Leal et al. (2023)
Etoposide	0.00	0.00	0.01	Beta	Leal et al. (2023)
Gemcitabine	0.02	0.02	0.03	Beta	Leal et al. (2023)
Nab-paclitaxel	0.29	0.22	0.36	Beta	Leal et al. (2023)
Paclitaxel	0.00	0.00	0.01	Beta	Leal et al. (2023)
Pemetrexed	0.07	0.05	0.09	Beta	Leal et al. (2023)
Vinorelbine	0.10	0.08	0.13	Beta	Leal et al. (2023)
Subsequent therapy proportion in the ST group					
Bevacizumab	0.11	0.08	0.14	Beta	Leal et al. (2023)
Carboplatin	0.14	0.11	0.18	Beta	Leal et al. (2023)
Cisplatin	0.03	0.02	0.04	Beta	Leal et al. (2023)
Crizotinib	0.06	0.05	0.08	Beta	Leal et al. (2023)
Erlotinib	0.25	0.19	0.31	Beta	Leal et al. (2023)
Etoposide	0.03	0.02	0.04	Beta	Leal et al. (2023)
Gemcitabine	0.03	0.02	0.04	Beta	Leal et al. (2023)
Nab-paclitaxel	0.25	0.19	0.31	Beta	Leal et al. (2023)
Paclitaxel	0.03	0.02	0.04	Beta	Leal et al. (2023)
Pemetrexed	0.14	0.11	0.18	Beta	Leal et al. (2023)
Vinorelbine	0.03	0.02	0.04	Beta	Leal et al. (2023)
SAE management cost (per event), $					
Anemia	2,048.76	1,536.57	2,560.95	Gamma	Insinga et al. (2018)
Pneumonia	7,176.87	5,382.65	8,971.09	Gamma	Insinga et al. (2018)
Leukopenia	461.50	346.13	576.88	Gamma	Shu et al. (2023)
Fatigue	859.64	644.73	1,074.55	Gamma	Insinga et al. (2018)
Dyspnoea	5,997.65	4,498.24	7,497.06	Gamma	Wong et al. (2018)
Pleural effusion	741.92	556.44	927.4	Gamma	Rothwell et al. (2021)
Musculoskeletal pain	4,232.54	3,174.41	5,290.68	Gamma	Ondhia et al. (2019)
Sepsis	17,477.67	13,108.25	21,847.09	Gamma	Li et al. (2022)
Utility					
PD	0.59	0.44	0.74	Beta	Chouaid et al. (2013)
PF	0.74	0.56	0.93	Beta	Chouaid et al. (2013)
Disutility					
Grade 1–2 AEs	0.01	0.0075	0.0125	Beta	Amdahl et al. (2016)
Grade 3–5 AEs	0.16	0.12	0.20	Beta	Amdahl et al. (2016)
Risk of AEs in the TTF + ST group					
Grade 1–2 AEs	0.38	0.29	0.48	Beta	Leal et al. (2023)
Grade 3–5 AEs	0.59	0.44	0.73	Beta	Leal et al. (2023)
Anemia	0.08	0.06	0.09	Beta	Leal et al. (2023)
Pneumonia	0.11	0.08	0.14	Beta	Leal et al. (2023)
Leukopenia	0.14	0.10	0.17	Beta	Leal et al. (2023)
Fatigue	0.04	0.03	0.05	Beta	Leal et al. (2023)
Dyspnoea	0.07	0.05	0.08	Beta	Leal et al. (2023)
Pleural effusion	0.02	0.017	0.028	Beta	Leal et al. (2023)
Musculoskeletal pain	0.03	0.02	0.04	Beta	Leal et al. (2023)
Sepsis	0.03	0.02	0.04	Beta	Leal et al. (2023)
Risk of SAEs in the ST group					
Grade 1–2 AEs	0.34	0.26	0.43	Beta	Leal et al. (2023)
Grade 3–5 AEs	0.56	0.42	0.70	Beta	Leal et al. (2023)
Anemia	0.08	0.06	0.10	Beta	Leal et al. (2023)
Pneumonia	0.11	0.08	0.14	Beta	Leal et al. (2023)
Leukopenia	0.14	0.11	0.18	Beta	Leal et al. (2023)
Fatigue	0.08	0.06	0.09	Beta	Leal et al. (2023)
Dyspnoea	0.03	0.02	0.04	Beta	Leal et al. (2023)
Pleural effusion	0.05	0.04	0.07	Beta	Leal et al. (2023)
Musculoskeletal pain	0.04	0.03	0.05	Beta	Leal et al. (2023)
Sepsis	0.04	0.03	0.05	Beta	Leal et al. (2023)

Abbr. OS, overall survival; PFS, progression-free survival; ND, not determined; AE, adverse event; ST, standard therapy; TTF, tumor treating fields; ICI, immune checkpoint inhibitor.

### Cost and utility data

In this study, we evaluated the cost-effectiveness from the perspective of the United States healthcare payers. We incorporated a wide range of cost sources, including cancer treatment, supportive care, laboratory tests and scans, managements of adverse events, intravenous administration, and hospitalization. To estimate the dose of agents, we assumed a typical 65-year-old patient with 70 kg in weight and 1.8 m^2^ in body surface area (BSA) (Liu et al., 2021). Drug costs were sourced from the Centers for Medicare and Medicaid Services or based on published articles, with adjustments made for inflation (Insinga et al., 2018; Wong et al., 2018; Criss et al., 2019; Ondhia et al., 2019; De Marzi et al., 2020; Dutt et al., 2020; Zhang et al., 2020; Rothwell et al., 2021; Gong et al., 2022; Li et al., 2022; Shen et al., 2022). Utilities for PFS and PD, scaling from 0 (death) to 1 (perfect health), and disutility of adverse events were derived from previously published studies (Chouaid et al., 2013; Amdahl et al., 2016). Costs and utilities were discounted at an annual rate of 5% (Smith and Gravelle, 2001) (Table 1).

### Sensitivity analysis

We performed one-way sensitivity analysis and probabilistic sensitivity analysis to evaluate the model’s robustness. In one-way sensitivity analysis, we varied the body weight, body surface area, cost, and proportion values by ±25%. Discount rates were varied between 0% and 8%, and utility/disutility values were adjusted by ±10%. For proportions with a baseline value of 0%, the lower and upper boundary were set to 0% and 1%, respectively. In probabilistic sensitivity analysis, we repeated 1,000 Monte Carlo iterations for all distributions assigned to costs (γ distribution), proportions (β distribution), utilities (β distribution), body surface area (normal distribution), body weight (normal distribution), and the discount rate (uniform distribution). The mean of the distributions was set to the baseline value, with the standard deviations set to make the boundaries of the 95% confidence intervals to approach the boundary values used in the one-way sensitivity analysis.

### Scenario analysis

Considering the costs and effects may vary greatly over time from the initiation of treatment, we further estimated cost-effectiveness of all the 6 treatments setting the time horizons to 5 years, 8 years, and 15 years, respectively.

## Results

### Base-case results

We constructed a Markov model and predicted the 10-year survival and treatment status of patients in the LUNAR study (Table 2). For the base case scenario, 10-year average costs of TTF + ST and ST alone were $434,969.3 and $130,571.3 per patient, while the corresponding QALYs were 1.809 and 1.124, respectively. The incremental costs and QALYs for TTF + ST compared with ST alone were $304,398.0 and 0.685, respectively, resulting in an ICER of $444,656.4 per QALY. Meanwhile, at a WTP threshold of $150,000 per QALY, the INMB of TTF + ST compared with ST alone was -$201,712.6.

**TABLE 2:** Base case results.

Treatment	Cost, $	Incremental cost, $	QALY	Incremental QALY	INMB^a^	ICER ($/QALY)
TTF + ST	434,969.3	304,398.0	1.809	0.685	−201,712.6	444,656.4
ST	130,571.3	NA	1.124	NA	NA	NA

^a^ At a willing-to-pay threshold at $150,000 per QALY, gained. Abbr. QALY, quality-adjusted life year; INMB, incremental net monetary benefit; ICER, incremental cost-effectiveness ratio; NA, not applicable; TTF, tumor treating fields; ST, standard therapy.

Furthermore, the subgroup analysis involving TTF + ICI, ICI alone, TTF + docetaxel, and docetaxel alone revealed that 10-year average costs for these treatments were $897,027.2, $238,404.7, $221,648.7, $92,599.1, respectively. The corresponding QALYs were 2.318, 1.399, 1.289, 1.035, respectively. Compared with the docetaxel group, the incremental costs for the TTF + ICI, ICI alone, and TTF + docetaxel group were $804,428.1, $145,805.6, and $129,049.6, and the incremental QALYs were 1.283, 0.364, and 0.253, which yielded ICERs of $627,315.5, $401,107.9, and $509,139.1 per QALY gained, and INMBs of -$612,078.0, -$91,279.5, and -$91,029.7, respectively. Moreover, compared with TTF + docetaxel, the incremental costs of TTF + ICI and ICI alone were $675,378.5 and $16,756.0, with the incremental QALYs of 1.029 and 0.11, respectively, which generated ICERs of $656,428.8 and $152,207.6 per QALY gained and INMBs of -$521,048.3 and -$249.9, respectively. Last, the incremental cost and incremental QALY of TTF + ICI compared with ICI alone were $658,622.5 and 0.919, yielding an ICER of $716,807.9 per QALY gained and an INMB of -$520,798.4 (Table 3).

**TABLE 3:** Subgroup analyses.

Treatment	Cost, $	Incremental cost, $	QALY	Incremental QALY	INMB^a^	ICER ($/QALY)	Compared with
TTF + ICI	897,027.2	804,428.1	2.318	1.283	−612,078.0	627,315.5	D
		658,622.5		0.919	−520,798.4	716,807.9	ICI
		675,378.5		1.029	−521,048.3	656,428.8	TTF + D
ICI	238,404.7	145,805.6	1.399	0.364	−91,279.5	401,107.9	D
		16,756.0		0.11	−249.9	152,207.6	TTF + D
TTF + D	221,648.7	129,049.6	1.289	0.253	−91,029.7	509,139.1	D
D	92,599.1	NA	1.035	NA	NA	NA	NA

^a^ At a willing-to-pay threshold at $150,000 per QALY, gained. Abbr. QALY, quality-adjusted life year; INMB, incremental net monetary benefit; ICER, incremental cost-effectiveness ratio; NA, not applicable.

### Sensitivity analyses

**FIGURE 2:**
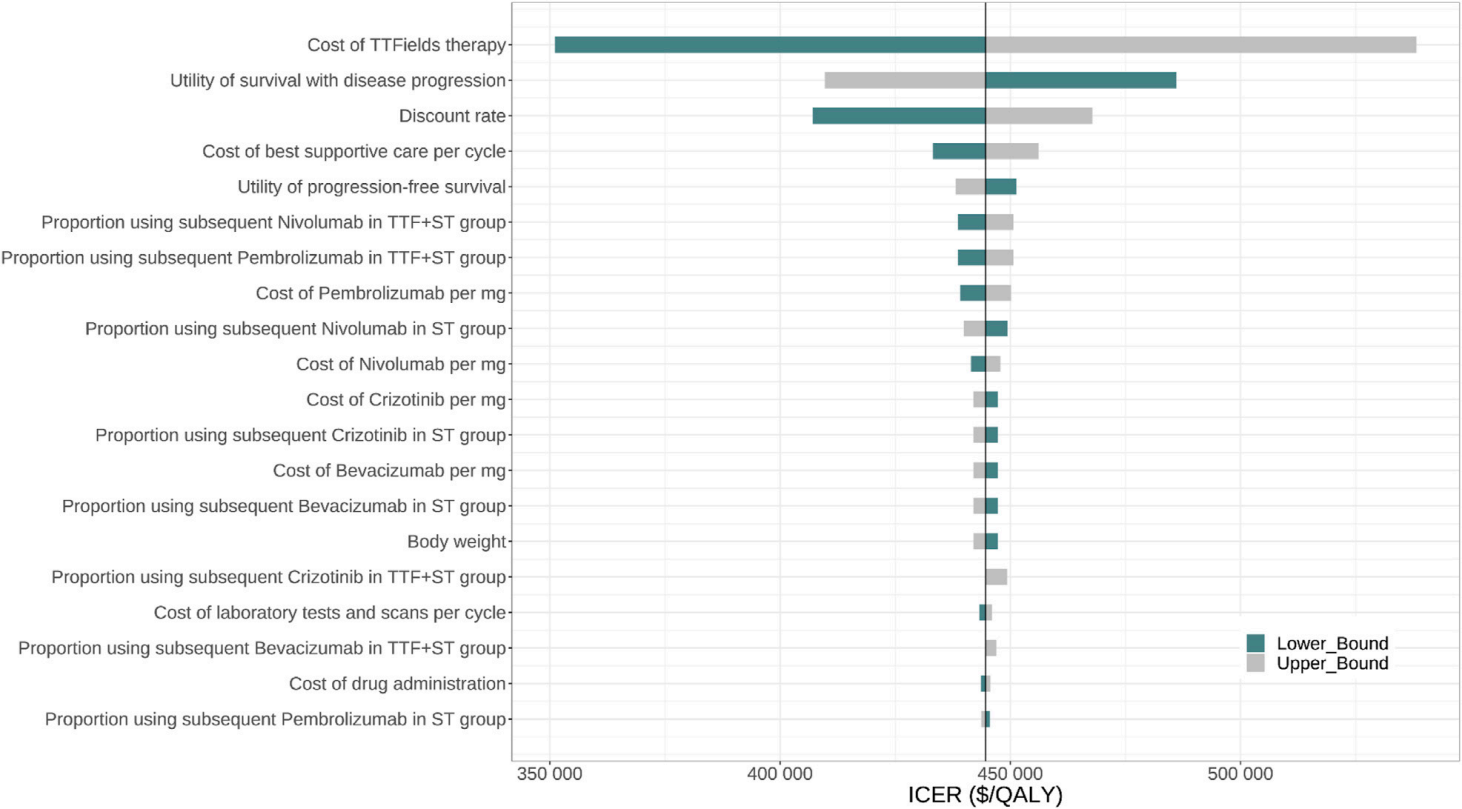
One-way sensitivity analysis for TTF + Standard therapy vs. Standard therapy. Abbr. TTF, tumor treating fields; ST, standard therapy; ICER, incremental cost-effectiveness ratio; QALY, quality-adjusted life-year.

**FIGURE 3:**
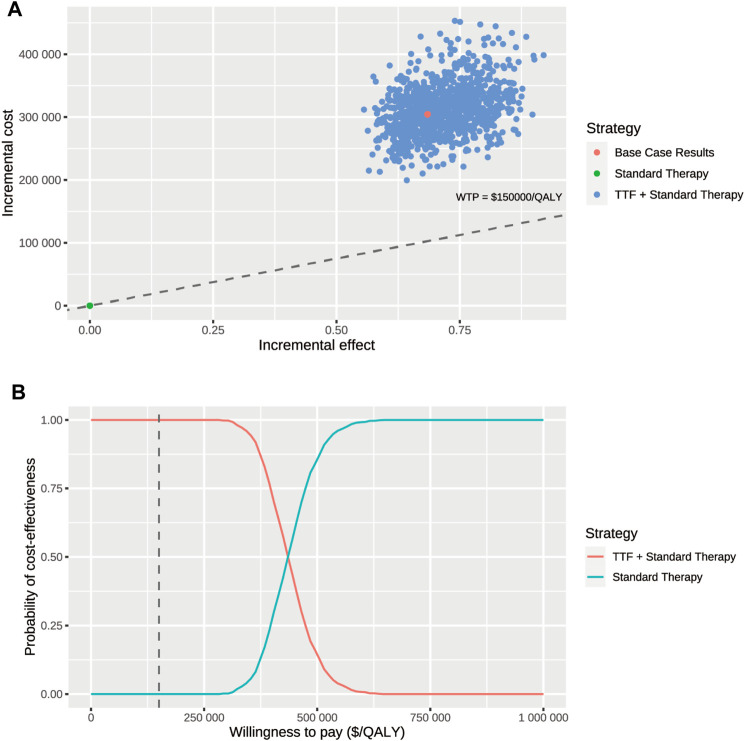
Probabilistic sensitivity analysis. **(A)** Incremental cost ($) and incremental effect (QALY) incurred by 1,000 probabilistic resamplings per strategy. **(B)** Probability of cost-effectiveness at varying willingness-to-pay. The dashed line represents the willing-to-pay threshold of $150,000 per QALY gained. Abbr. TTF, tumor-treating fields; WTP, willingness-to-pay.

Figure 2 displayed the 20 most influential factors identified in the one-way sensitivity analysis. The cost of TTF per month emerged as the top factor influencing the ICER. As the cost varied between its lower boundary ($20,410.86) and upper boundary ($34,018.1), the ICER fluctuated from $351,099.2 to $538,213.6 per QALY gained. The ICER was also sensitive to the utility of survival with disease progression (ICER ranging from $486,052.7 to $409,757.0 per QALY gained), discount rate (ICER ranging from $407,087.6 to $467,814.7 per QALY gained), the cost of best supportive care per cycle (ICER ranging from $433,225.9 to $456,086.9 per QALY gained), and the utility of progression-free survival (ICER ranging from $451,341.0 to $438,166.9 per QALY gained). Furthermore, we assigned a range of $0 to $30,000 for the cost of TTF per month to evaluate the corresponding INMB of TTF + ST versus ST alone at the WTP threshold of $150,000 per QALY gained, and the results showed that the INMB became positive when the cost of TTF per month was below $5,787 (Supplementary Figure S5A). According to the probabilistic sensitivity analysis (Figure 3), the total cost of TTF + ST ranged from $333,554.6 to $589,389.5, while that of ST alone ranged from $96,268.2 to $186,130.7. The QALYs of them ranged from 1.513 to 2.173 and from 0.947 to 1.299, respectively. Accordingly, all the resamples yielded positive ICER, ranging from $292,215.0 to $638,461.3 per QALY gained, which indicated that the probability of TTF + ST being cost-effective was 0% at the WTP threshold of $150,000 per QALY gained.

In terms of the subgroups, we conducted one-way sensitivity analyses for all 6 pairwise comparisons, including TTF + ICI vs. docetaxel alone, TTF + docetaxel vs. docetaxel alone, TTF + ICI vs. TTF + docetaxel, ICI alone vs. TTF + docetaxel, and ICI alone vs. docetaxel alone, the results of which were demonstrated in the Supplementary Figures S4A–F. In the first five comparisons, the cost of TTF per month remained the most influential factor in determining the ICERs, while in the comparison of ICI alone vs. docetaxel alone, the most influential factor was the cost of nivolumab per mg (Supplementary Figure S4F). We also conducted a probabilistic sensitivity analysis for the 4 treatments. The results showed that the probabilities of TTF + ICI, TTF + docetaxel, and ICI alone being cost-effective were 0% at a WTP threshold of $150,000 per QALY gained (Supplementary Figures S4G, H). Similarly, we explored the influences of the cost of TTF per month on the INMBs of TTF + ICI versus docetaxel, TTF + ICI versus ICI, TTF + docetaxel versus docetaxel, and ICI versus TTF + docetaxel. The results showed that the INMB of TTF + docetaxel versus docetaxel became positive when the cost of TTF per month was below $8,381, and the INMB of ICI versus TTF + docetaxel became negative when the cost was below $27,266 per month (Supplementary Figure S4B). Notably, the INMB of TTF + ICI versus docetaxel and that of TTF + ICI versus ICI stayed negative even if the cost of TTF was $0 per month (Supplementary Figure S4B).

### Scenario analysis

The base case results were based on a 10-year time frame. To investigate the effect of setting the time horizon in affecting cost-effectiveness. We further assessed the cost-effectiveness of all the 6 treatments in 5-year, 8-year, and 15-year time horizons. Specifically, the ICER of TTF + ST compared with ST alone decreased from $559933.7 per QALY gained (5 years) to $467513.0 per QALY gained (8 years) and then to $420263.5 per QALY gained (15 years). Besides, the ICER of TTF + ICI vs. docetaxel, the ICER of TTF + ICI vs. TTF + docetaxel, the ICER of ICI vs. docetaxel, and the ICER of TTF + docetaxel vs. docetaxel also decreased but were still above the WTP threshold of $150,000 per QALY gained, as the time horizon extended. It was also noteworthy that ICI stayed cost-effective compared with TTF + docetaxel in 5-year, and 8-year time horizons.

## Discussion

First-line therapies for patients with driver gene-negative metastatic NSCLC include ICI monotherapy (high PD-L1 expression) (Mok et al., 2019; Herbst et al., 2020; Papadimitrakopoulou et al., 2020; Akinboro et al., 2022), ICI + platinum-based chemotherapy (regardless of PD-L1 expression) (Reck et al., 2022), and a four-drug combination of bevacizumab and atezolizumab plus chemotherapy (Socinski et al., 2018). While platinum-based first-line treatment has shown certain benefits, disease progression remains a common occurrence (Ramlau et al., 2012). Therefore, finding new therapy is crucial to improve patient outcomes. The groundbreaking LUNAR study marks the first instance where TTF + ST has exhibited extended survival in patients with metastatic NSCLC who have previously experienced failure with platinum-based therapy. However, the cost-effectiveness of this treatment, which is essential in its clinical application, is yet to be explored. Our study demonstrates that although TTF + ST leads to an average gain of 0.685 QALY compared with ST alone, it is not a cost-effective treatment for patients with NSCLC after platinum-based therapy in the United States, and the cost of TTF per month is the primary factor influencing its cost-effectiveness.

The lack of cost-effectiveness in combining TTF with standard therapy is attributed to the high cost of TTF rather than the limited health benefits. The high cost of TTF treatment is a primary concern in its cost-effectiveness, and several studies have assessed the cost-effectiveness of TTF + temozolomide (TMZ) in newly diagnosed glioblastoma (Bernard-Arnoux et al., 2016; Connock et al., 2019; Guzauskas et al., 2019). From the perspective of French health insurance, Bernard-Arnoux et al. (2016) demonstrated that the ICER of TTF + TMZ versus TMZ was €596 411 per life-year gained (LYG), and the probabilistic sensitivity analysis indicated that TTF + TMZ had 0% chance to be cost-effective at a WTP threshold of €100,000/LYG. Connock et al. (2019) reached a similar conclusion through a partitioned survival model in French. The ICER of TTF + TMZ compared with TMZ alone was €510,273/LYG, which widely exceeded the WTP threshold of €100,000/LYG. To achieve an ICER of less than €100,000/LYG, the cost of TTF device needs to be reduced by approximately 85%. However, from the perspective of the United States health insurance with a 5-year time horizon, Guzauskas et al. (2019) demonstrated that TTF + TMZ, with an ICER of $197,336 per QALY gained was cost-effective within the WTP threshold of $200,000 per QALY gained in the United States. These studies collectively emphasize that the high cost of TTF significantly impacts the ICER (Bernard-Arnoux et al., 2016; Connock et al., 2019; Guzauskas et al., 2019). Similarly, our analysis reveals that, in the treatment of advanced NSCLC, the TTF cost has the most substantial influence on the ICER of TTF + ST versus ST alone and the ICERs of subgroup comparisons involving TTF. Further analysis demonstrates that TTF + ST may only be cost-effective when the price of TTF per month is significantly reduced to less than $5,787, which represents about 21.3% of the estimated cost, $27,214,48 per month, in the base case analysis.

Looking into the two types of ST, ICI and docetaxel, our subgroup analyses showed that neither TTF + ICI nor TTF + docetaxel is cost-effective compared with either ICI alone or docetaxel alone, and ICI alone is cost-effective compared with TTF + docetaxel. The sensitivity analysis also identified the cost of TTF per month as the primary target to enhance the cost-effectiveness of TTF + ICI and TTF + docetaxel. It is noteworthy that the substantial increase in patients’ average QALY (2.318) in the TTF + ICI group compared to that of the ICI group (1.399), the TTF + docetaxel group (1.289), and the docetaxel group (1.035) emphasizes that TTF + ICI is an effective strategy for patients with advanced NSCLC. Moreover, in the comparison of TTF + ICI versus TTF + docetaxel and the comparison of TTF + ICI versus docetaxel alone, the costs of the ICIs (nivolumab, atezolizumab, and pembrolizumab) are among the top 6 most influential factors determining the ICERs, indicating that, besides the cost of TTF, the high costs of ICIs are key factors impairing the cost-effectiveness of TTF + ICI. Previous studies have consistently indicated a lack of cost-effectiveness of several ICIs in second-line treatment of advanced NSCLC in Swiss, the United States, Australia, or China, and suggested that the cost of these ICIs was essential in affecting the cost-effectiveness (Matter-Walstra et al., 2016; Aguiar et al., 2017; Gao and Li, 2019; Liu et al., 2020). Therefore, cutting down the prices of both TTF and immune checkpoint inhibitors is a possible way to generate economic benefits (Qiao et al., 2021; Borghini et al., 2022; Liu et al., 2022; Wang et al., 2022; Zheng et al., 2022; Cheng et al., 2023; Liang et al., 2023).

Our study also has certain limitations. Firstly, the use of published K-M curves instead of individual patient data from the LUNAR study introduces a potential source of inaccuracy when reconstructing the clinical data, but K-M curves were recreated to ensure the accuracy on the image level. Additionally, given the lack of manufacturer pricing data, the estimated price of TTF was based on inflation from previously published literature, which may be overestimated. However, the impact of TTF cost on the cost-effectiveness was intendedly explored in our study by adopting a wide range of TTF cost per month. Furthermore, our study tried to resemble the design of LUNAR study to ensure the coherence of clinical parameters, but real-life therapeutic situation may differ from the trial, such as different proportion of patients receiving ICI versus patients receiving docetaxel and different dosage of agents, which may lead to a lack of generalizability of the results. Finally, we used disutility from a study on renal cell carcinoma due to a lack of disutility data source of grade 1–2 or grade 3–5 adverse events, which can lead to inaccuracies in QALYs (Amdahl et al., 2016).

## Conclusion

From the perspective of the United States insurance payers, TTF + ST does not appear to be a cost-effective therapeutic approach for metastatic NSCLC. The predominant factor affecting the potential of financial benefit is the cost of TTF. Therefore, strict regulation of TTF pricing by health administrations is essential to increase the accessibility and affordability for more patients with advanced NSCLC.

## Data Availability

The original contributions presented in the study are included in the article/Supplementary Material, further inquiries can be directed to the corresponding authors.

## Abbreviations

QALYs, quality-adjusted life-years; ICER, incremental cost-effectiveness ratio; WTP, willingness-to-pay; NMB, net monetary benefit.
