# Supplementary material for: Cost-effectiveness of tumor-treating fields plus standard therapy for advanced non-small cell lung cancer progressed after platinum-based therapy in the United States
Source: Front Pharmacol. 2024 Feb 5;15:1333128. doi: 10.3389/fphar.2024.1333128 (PMC10875105; doi:10.3389/fphar.2024.1333128)
Supplement: Supplementary file 1 [file DataSheet1.docx]

Supplementary Material

**Table S1.** Summary of AIC and BIC Scores for Parametric Models.

| **Scores** | **Exponential** | **Weibull** | **Loglogistic** | **Lognormal** | **Generalized gamma** | **Gamma** | **Gompertz** | **Royston/Parmar (k=0)** | **Royston/Parmar (k=1)** | **Royston/Parmar (k=2)** |
| --- | --- | --- | --- | --- | --- | --- | --- | --- | --- | --- |
| **OS of TTF + ST** | | | | | | | | | | |
| AIC | 742.70 | 743.73 | 740.01 | 752.56 | 744.64 | 744.19 | 739.83 | 743.73 | 745.51 | 737.87 |
| BIC | 745.62 | 749.57 | 745.85 | 758.40 | 753.40 | 750.03 | 745.67 | 749.57 | 754.27 | 749.55 |
| **OS of ST** | | | | | | | | | | |
| AIC | 826.71 | 828.26 | 833.13 | 837.19 | 829.98 | 828.15 | 828.38 | 828.26 | 829.71 | 831.65 |
| BIC | 829.64 | 834.13 | 838.99 | 843.06 | 838.79 | 834.02 | 834.25 | 834.13 | 838.51 | 843.38 |
| **PFS of TTF + ST** | | | | | | | | | | |
| AIC | 629.59 | 630.65 | 614.81 | 611.22 | 608.90 | 628.57 | 629.95 | 630.65 | 613.69 | 614.70 |
| BIC | 632.51 | 636.49 | 620.65 | 617.06 | 617.65 | 634.41 | 635.79 | 636.49 | 622.45 | 626.38 |
| **PFS of ST** | | | | | | | | | | |
| AIC | 674.78 | 676.55 | 653.45 | 649.91 | 643.27 | 674.69 | 672.53 | 676.55 | 647.06 | 645.22 |
| BIC | 677.72 | 682.42 | 659.32 | 655.78 | 652.07 | 680.56 | 678.40 | 682.42 | 655.86 | 656.96 |
| **OS of TTF + Docetaxel** | | | | | | | | | | |
| AIC | 403.98 | 405.80 | 402.32 | 406.47 | 405.85 | 405.40 | 405.02 | 405.80 | 406.81 | 405.59 |
| BIC | 406.24 | 410.32 | 406.85 | 410.99 | 412.63 | 409.93 | 409.54 | 410.32 | 413.60 | 414.64 |
| **OS of Docetaxel** | | | | | | | | | | |
| AIC | 409.01 | 408.72 | 406.90 | 406.66 | 408.11 | 407.87 | 410.50 | 408.72 | 407.87 | 409.66 |
| BIC | 411.27 | 413.24 | 411.42 | 411.19 | 414.89 | 412.39 | 415.03 | 413.24 | 414.66 | 418.71 |
| **PFS of TTF + Docetaxel** | | | | | | | | | | |
| AIC | 327.97 | 319.23 | 316.21 | 314.54 | 316.64 | 326.52 | 316.39 | 319.23 | 317.95 | 319.53 |
| BIC | 330.23 | 323.75 | 320.74 | 319.06 | 321.16 | 331.04 | 323.18 | 323.75 | 324.74 | 328.58 |
| **PFS of Docetaxel** | | | | | | | | | | |
| AIC | 333.56 | 333.04 | 322.95 | 323.38 | 330.69 | 335.49 | 325.20 | 333.04 | 325.08 | 326.24 |
| BIC | 335.82 | 337.56 | 327.48 | 327.91 | 335.22 | 340.01 | 331.99 | 337.56 | 331.86 | 335.29 |
| **OS of TTF + ICI** | | | | | | | | | | |
| AIC | 293.51 | 294.83 | 288.80 | 285.99 | 282.12 | 295.37 | 290.84 | 294.83 | 285.76 | 283.51 |
| BIC | 295.70 | 299.21 | 293.18 | 290.37 | 288.69 | 299.76 | 295.22 | 299.21 | 292.33 | 292.27 |
| **OS of ICI** | | | | | | | | | | |
| AIC | 410.67 | 412.63 | 413.71 | 412.58 | 412.56 | 412.67 | 413.71 | 412.63 | 413.88 | 415.57 |
| BIC | 412.89 | 417.07 | 418.15 | 417.02 | 416.99 | 417.11 | 420.37 | 417.07 | 420.53 | 424.45 |
| **PFS of TTF + ICI** | | | | | | | | | | |
| AIC | 293.51 | 294.83 | 288.80 | 285.99 | 282.12 | 295.38 | 290.84 | 294.83 | 285.76 | 283.51 |
| BIC | 295.70 | 299.21 | 293.18 | 290.37 | 288.69 | 299.76 | 295.22 | 299.21 | 292.33 | 292.27 |
| **PFS of ICI** | | | | | | | | | | |
| AIC | 341.72 | 343.31 | 331.77 | 328.59 | 343.72 | 338.52 | 305.11 | 343.31 | 326.39 | 314.83 |
| BIC | 343.94 | 347.75 | 336.21 | 333.03 | 348.16 | 342.96 | 311.77 | 347.75 | 333.04 | 323.71 |

***Abbr.*** *AIC, Akaike information criterion; BIC, Bayesian information criterion; OS, overall survival; PFS, progression-free survival; TTF, Tumor Treating Fields; ST, standard therapy; ICI, immune checkpoint inhibitor.*

**Table S2.** Additional Parameters Input to the Model for the subgroup analysis.

| **Parameters** | **Base Value** | **Range** | | **Distribution** | **Source** |
| --- | --- | --- | --- | --- | --- |
|  |  | **Minimum** | **Maximum** |  |  |
| **Clinical** | | | | | |
| Loglogistic model for OS of TTF + ICI | Shape: 1.185 | ND | ND | ND | Model fitting |
|  | Scale: 17.909 |  |  |  |  |
| Exponential model for OS of ICI | Rate: 0.062 | ND | ND | ND | Model fitting |
| Loglogistic model for OS of TTF + Docetaxel | Shape: 1.526 | ND | ND | ND | Model fitting |
|  | Scale: 10.601 |  |  |  |  |
| Lognormal model for OS of Docetaxel | Log-mean: 2.157 | ND | ND | ND | Model fitting |
|  | Log-sd: 1.047 |  |  |  |  |
| Generalized gamma model for PFS of TTF+ ICI | Mu: 0.968 | ND | ND | ND | Model fitting |
|  | Sigma: 1.15 |  |  |  |  |
|  | Q: -1.602 |  |  |  |  |
| Generalized gamma model for PFS of ICI | Mu: 0.322 | ND | ND | ND | Model fitting |
|  | Sigma: 0.421 |  |  |  |  |
|  | Q: -3.657 |  |  |  |  |
| Lognormal model for PFS of TTF+ Docetaxel | Log-mean: 1.395 | ND | ND | ND | Model fitting |
|  | Log-sd: 0.805 |  |  |  |  |
| Loglogistic model for PFS of Docetaxel | Shape: 1.87 | ND | ND | ND | Model fitting |
|  | Scale: 4.057 |  |  |  |  |
| **Subsequent therapy proportion in the TTF + ICI group** | | | | | |
| Bevacizumab | 0 | 0 | 0.01 | Beta | LUNAR |
| Carboplatin | 0.22 | 0.17 | 0.28 | Beta | LUNAR |
| Cisplatin | 0 | 0 | 0.01 | Beta | LUNAR |
| Crizotinib | 0 | 0 | 0.01 | Beta | LUNAR |
| Docetaxel | 0.61 | 0.46 | 0.76 | Beta | LUNAR |
| Erlotinib | 0 | 0 | 0.01 | Beta | LUNAR |
| Etoposide | 0 | 0 | 0.01 | Beta | LUNAR |
| Gemcitabine | 0.17 | 0.13 | 0.21 | Beta | LUNAR |
| Nab-paclitaxel | 0 | 0 | 0.01 | Beta | LUNAR |
| Nivolumab | 0.11 | 0.08 | 0.14 | Beta | LUNAR |
| Paclitaxel | 0.11 | 0 | 0.01 | Beta | LUNAR |
| Pembrolizumab | 0.06 | 0.17 | 0.28 | Beta | LUNAR |
| Pemetrexed | 0.06 | 0 | 0.01 | Beta | LUNAR |
| Vinorelbine | 0 | 0 | 0.01 | Beta | LUNAR |
| **Subsequent therapy proportion in the ICI group** | | | | | |
| Bevacizumab | 0.13 | 0.1 | 0.16 | Beta | LUNAR |
| Carboplatin | 0.25 | 0.19 | 0.31 | Beta | LUNAR |
| Cisplatin | 0 | 0 | 0.01 | Beta | LUNAR |
| Crizotinib | 0.06 | 0.05 | 0.08 | Beta | LUNAR |
| Docetaxel | 0.56 | 0.42 | 0.70 | Beta | LUNAR |
| Erlotinib | 0 | 0 | 0.01 | Beta | LUNAR |
| Etoposide | 0 | 0 | 0.01 | Beta | LUNAR |
| Gemcitabine | 0.19 | 0.14 | 0.24 | Beta | LUNAR |
| Nab-paclitaxel | 0.06 | 0.05 | 0.08 | Beta | LUNAR |
| Nivolumab | 0.06 | 0.05 | 0.08 | Beta | LUNAR |
| Paclitaxel | 0.06 | 0.10 | 0.16 | Beta | LUNAR |
| Pembrolizumab | 0 | 0.19 | 0.31 | Beta | LUNAR |
| Pemetrexed | 0.13 | 0 | 0.01 | Beta | LUNAR |
| Vinorelbine | 0.06 | 0.05 | 0.08 | Beta | LUNAR |
| **Subsequent therapy proportion in the TTF + docetaxel group** | | | | | |
| Bevacizumab | 0 | 0 | 0.01 | Beta | LUNAR |
| Carboplatin | 0.13 | 0.10 | 0.16 | Beta | LUNAR |
| Cisplatin | 0.04 | 0.03 | 0.05 | Beta | LUNAR |
| Crizotinib | 0 | 0 | 0.01 | Beta | LUNAR |
| Docetaxel | 0.17 | 0.13 | 0.21 | Beta | LUNAR |
| Erlotinib | 0 | 0 | 0.01 | Beta | LUNAR |
| Etoposide | 0.04 | 0.03 | 0.05 | Beta | LUNAR |
| Gemcitabine | 0.39 | 0.29 | 0.49 | Beta | LUNAR |
| Nab-paclitaxel | 0 | 0 | 0.01 | Beta | LUNAR |
| Nivolumab | 0.04 | 0.03 | 0.05 | Beta | LUNAR |
| Paclitaxel | 0.09 | 0 | 0.01 | Beta | LUNAR |
| Pembrolizumab | 0.09 | 0.10 | 0.16 | Beta | LUNAR |
| Pemetrexed | 0.09 | 0.03 | 0.05 | Beta | LUNAR |
| Vinorelbine | 0.17 | 0 | 0.01 | Beta | LUNAR |
| **Subsequent therapy proportion in the docetaxel group** | | | | | |
| Bevacizumab | 0.1 | 0.08 | 0.13 | Beta | LUNAR |
| Carboplatin | 0.05 | 0.04 | 0.06 | Beta | LUNAR |
| Cisplatin | 0.05 | 0.04 | 0.06 | Beta | LUNAR |
| Crizotinib | 0.05 | 0.04 | 0.06 | Beta | LUNAR |
| Docetaxel | 0 | 0 | 0.01 | Beta | LUNAR |
| Erlotinib | 0.05 | 0.04 | 0.06 | Beta | LUNAR |
| Etoposide | 0.05 | 0.04 | 0.06 | Beta | LUNAR |
| Gemcitabine | 0.3 | 0.23 | 0.38 | Beta | LUNAR |
| Nab-paclitaxel | 0 | 0 | 0.01 | Beta | LUNAR |
| Nivolumab | 0.2 | 0.15 | 0.25 | Beta | LUNAR |
| Paclitaxel | 0 | 0.08 | 0.13 | Beta | LUNAR |
| Pembrolizumab | 0.05 | 0.04 | 0.06 | Beta | LUNAR |
| Pemetrexed | 0.15 | 0.04 | 0.06 | Beta | LUNAR |
| Vinorelbine | 0.15 | 0.04 | 0.06 | Beta | LUNAR |
| **Risk of AEs in the TTF+ICI group** |  |  |  |  |  |
| Grade 1-2 AEs | 0.43 | 0.32 | 0.54 | Beta | LUNAR |
| Grade 3-5 AEs | 0.55 | 0.41 | 0.69 | Beta | LUNAR |
| Anemia | 0.07 | 0.05 | 0.09 | Beta | LUNAR |
| Pneumonia | 0.06 | 0.05 | 0.08 | Beta | LUNAR |
| Leukopenia | 0.03 | 0.02 | 0.04 | Beta | LUNAR |
| Fatigue | 0.03 | 0.02 | 0.04 | Beta | LUNAR |
| Dyspnoea | 0.03 | 0.02 | 0.04 | Beta | LUNAR |
| Pleural effusion | 0.01 | 0.01 | 0.01 | Beta | LUNAR |
| Musculoskeletal pain | 0.01 | 0.01 | 0.01 | Beta | LUNAR |
| Sepsis | 0.01 | 0.01 | 0.01 | Beta | LUNAR |
| **Risk of AEs in the ICI group** | | | | | |
| Grade 1-2 AEs | 0.42 | 0.32 | 0.53 | Beta | LUNAR |
| Grade 3-5 AEs | 0.48 | 0.36 | 0.60 | Beta | LUNAR |
| Anemia | 0.03 | 0.02 | 0.04 | Beta | LUNAR |
| Pneumonia | 0.11 | 0.08 | 0.14 | Beta | LUNAR |
| Leukopenia | 0.03 | 0.02 | 0.04 | Beta | LUNAR |
| Fatigue | 0.03 | 0.02 | 0.04 | Beta | LUNAR |
| Dyspnoea | 0.02 | 0.02 | 0.03 | Beta | LUNAR |
| Pleural effusion | 0.03 | 0.02 | 0.04 | Beta | LUNAR |
| Musculoskeletal pain | 0.05 | 0.04 | 0.06 | Beta | LUNAR |
| Sepsis | 0.05 | 0.04 | 0.06 | Beta | LUNAR |
| **Risk of AEs in the TTF + docetaxel group** | | | | | |
| Grade 1-2 AEs | 0.33 | 0.25 | 0.41 | Beta | LUNAR |
| Grade 3-5 AEs | 0.62 | 0.47 | 0.78 | Beta | LUNAR |
| Anemia | 0.08 | 0.06 | 0.10 | Beta | LUNAR |
| Pneumonia | 0.17 | 0.13 | 0.21 | Beta | LUNAR |
| Leukopenia | 0.24 | 0.18 | 0.30 | Beta | LUNAR |
| Fatigue | 0.05 | 0.04 | 0.06 | Beta | LUNAR |
| Dyspnoea | 0.11 | 0.08 | 0.14 | Beta | LUNAR |
| Pleural effusion | 0.03 | 0.02 | 0.04 | Beta | LUNAR |
| Musculoskeletal pain | 0.05 | 0.04 | 0.06 | Beta | LUNAR |
| Sepsis | 0.05 | 0.04 | 0.06 | Beta | LUNAR |
| **Risk of SAEs in the docetaxel group** |  |  |  |  |  |
| Grade 1-2 AEs | 0.26 | 0.2 | 0.33 | Beta | LUNAR |
| Grade 3-5 AEs | 0.63 | 0.47 | 0.79 | Beta | LUNAR |
| Anemia | 0.13 | 0.1 | 0.16 | Beta | LUNAR |
| Pneumonia | 0.12 | 0.09 | 0.15 | Beta | LUNAR |
| Leukopenia | 0.25 | 0.19 | 0.31 | Beta | LUNAR |
| Fatigue | 0.12 | 0.09 | 0.15 | Beta | LUNAR |
| Dyspnoea | 0.04 | 0.03 | 0.05 | Beta | LUNAR |
| Pleural effusion | 0.07 | 0.05 | 0.09 | Beta | LUNAR |
| Musculoskeletal pain | 0.03 | 0.02 | 0.04 | Beta | LUNAR |
| Sepsis | 0.03 | 0.02 | 0.04 | Beta | LUNAR |

***Abbr.*** *OS = overall survival; PFS = progression-free survival; ND = not determined; AE = adverse event; ST = standard therapy; TTF = tumor treating fields; ICI = immune checkpoint inhibitor.*

**Table S3.** Scenario analyses.

| **Treatment/Scenarios** | **Cost, $** | **Incremental Cost, $** | **QALY** | **Incremental QALY** | **NMB*** | **ICER ($/QALY)** | **Comparator** |
| --- | --- | --- | --- | --- | --- | --- | --- |
| **Time horizon = 5 years** |  |  |  |  |  |  |  |
| TTF + ST | 382842.0 | 258809.6 | 1.519 | 0.462 | -189477.4 | 559933.7 | ST |
| ST | 124032.4 | NA | 1.057 | NA | NA | NA | NA |
| TTF + ICI | 697853.9 | 611081.2 | 1.881 | 0.892 | -477242.7 | 684871.6 | D |
|  |  | 494895.9 |  | 0.672 | -394082.8 | 736356.3 | ICI |
|  |  | 488619.1 |  | 0.720 | -380662.1 | 678908.1 | TTF + D |
| ICI | 202958.0 | 116185.3 | 1.209 | 0.220 | -83160 | 527709.5 | D |
|  |  | -6276.8 |  | 0.048 | 13420.7 | dominate | TTF + D |
| TTF + D | 209234.9 | 122462.2 | 1.161 | 0.173 | -96580.6 | 709746.6 | D |
| D | 86772.7 | NA | 0.989 | NA | NA | NA | NA |
| **Time horizon = 8 years** |  |  |  |  |  |  |  |
| TTF + ST | 421278.1 | 292432.7 | 1.732 | 0.626 | -198606.7 | 467513.0 | ST |
| ST | 128845.4 | NA | 1.106 | NA | NA | NA | NA |
| TTF + ICI | 836888.6 | 745377.6 | 2.196 | 1.169 | -569970.8 | 637413.2 | D |
|  |  | 609141.8 |  | 0.854 | -481056.2 | 713361.0 | ICI |
|  |  | 618526.8 |  | 0.941 | -477408.4 | 657455.2 | TTF + D |
| ICI | 227746.9 | 136235.9 | 1.342 | 0.315 | -88914.6 | 431843.5 | D |
|  |  | 9385.1 |  | 0.087 | 3647.7 | 108016.6 | TTF + D |
| TTF + D | 218361.8 | 126850.8 | 1.255 | 0.229 | -92562.3 | 554928.0 | D |
| D | 91510.9 | NA | 1.026 | NA | NA | NA | NA |
| **Time horizon = 15 years** |  |  |  |  |  |  |  |
| TTF + ST | 453242.6 | 320294.7 | 1.911 | 0.762 | -205975.5 | 420263.5 | ST |
| ST | 132947.9 | NA | 1.148 | NA | NA | NA | NA |
| TTF + ICI | 996867.9 | 903325.2 | 2.518 | 1.474 | -682270.8 | 612965.6 | D |
|  |  | 741282.3 |  | 1.027 | -587282.9 | 722031.0 | ICI |
|  |  | 770297.5 |  | 1.178 | -593629 | 654019.4 | TTF + D |
| ICI | 255585.6 | 162043.0 | 1.491 | 0.447 | -94987.9 | 362484.9 | D |
|  |  | 29015.2 |  | 0.151 | -6346.1 | 191992.1 | TTF + D |
| TTF + D | 226570.4 | 133027.7 | 1.340 | 0.296 | -88641.8 | 449560.1 | D |
| D | 93542.6 | NA | 1.044 | NA | NA | NA | NA |

*At a willing-to-pay threshold at $150,000 per QALY gained.

***Abbr.*** *QALY = quality-adjusted life year; INMB = incremental net monetary benefit; ICER = incremental cost-effectiveness ratio; TTF = tumor treating fields; ICI = immune checkpoint inhibitor; D = docetaxel; ST = standard therapy; NA: not applicable.*

A)


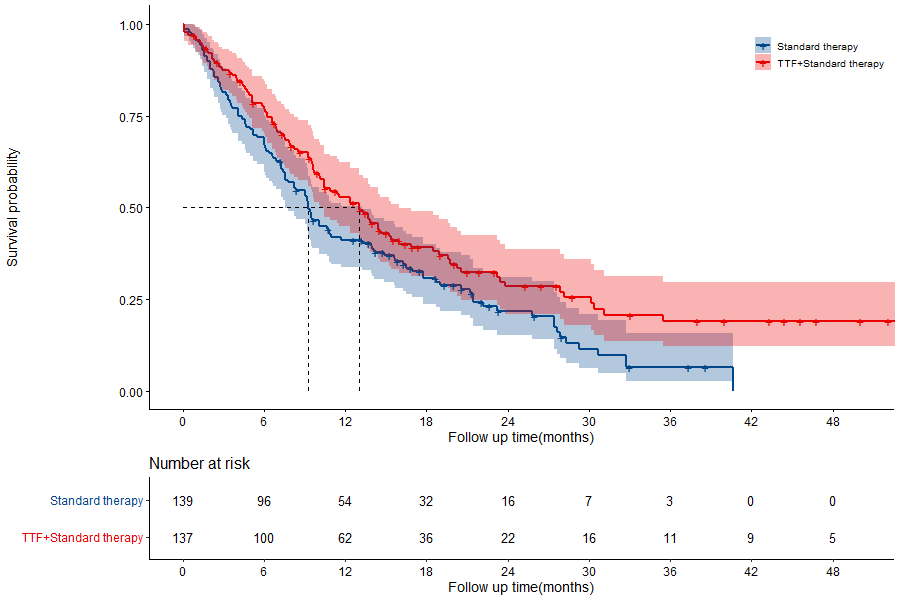


B)


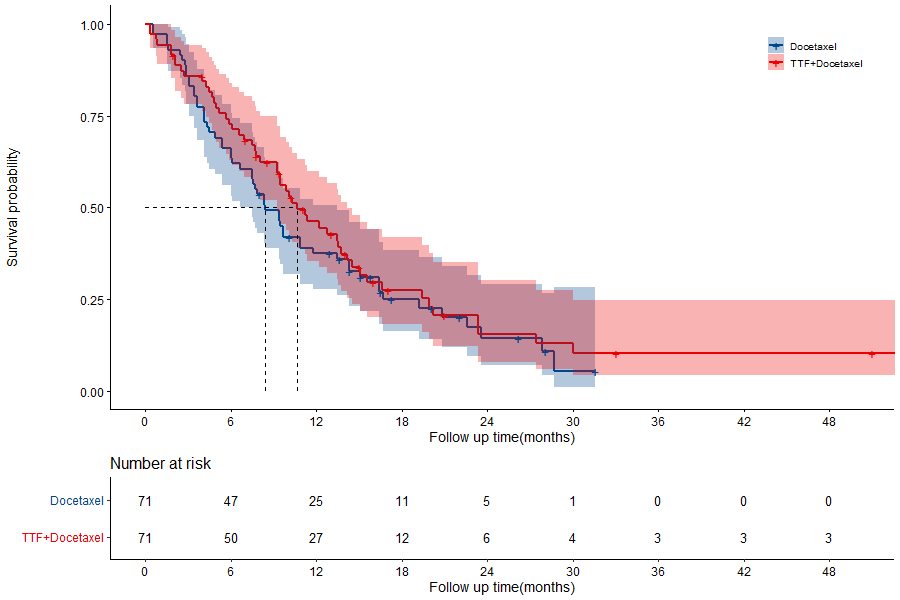


C)

.
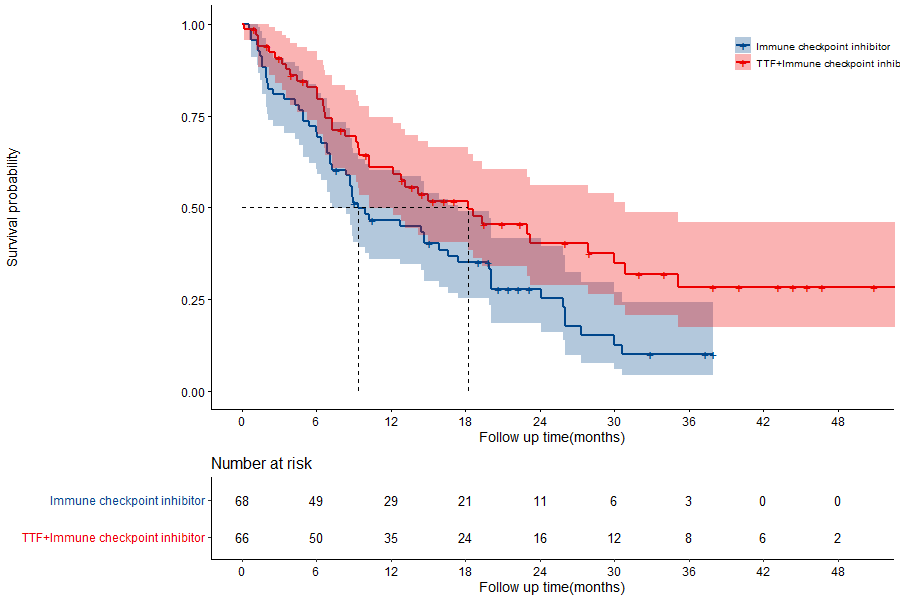


D)


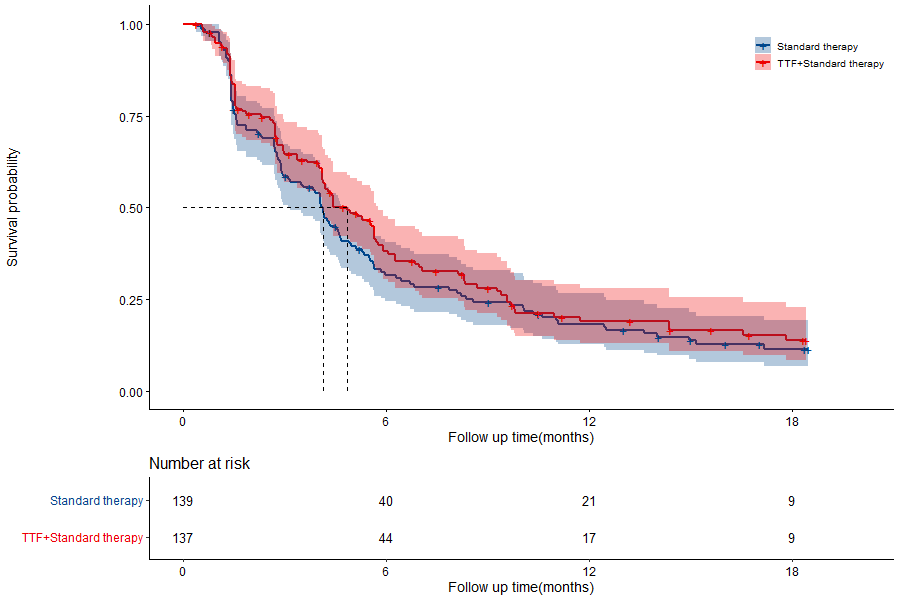


E)

**
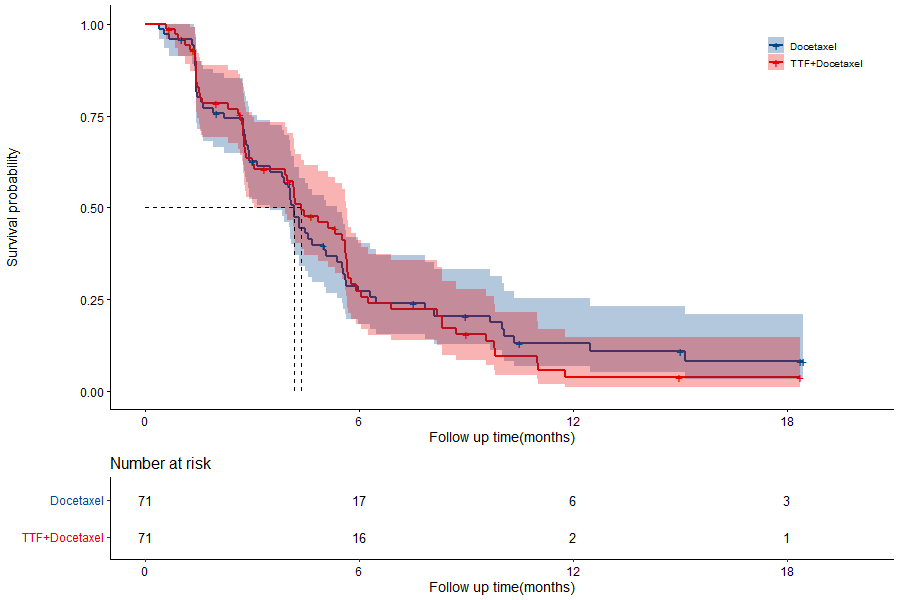
**

F)


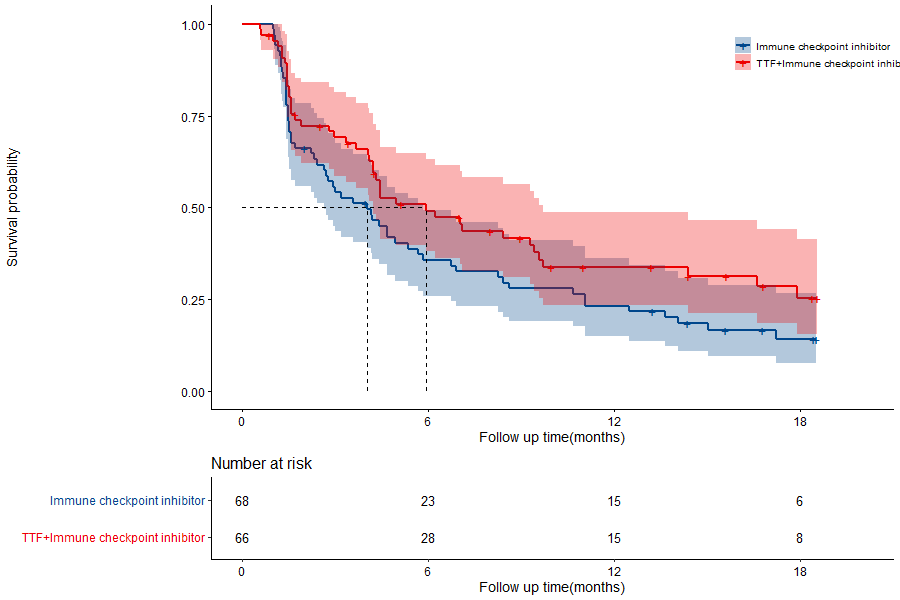


**Figure S1.** Replicated (A-C) overall survival and (D-F) progression-free survival Kaplan-Meier curves using reconstructed individual patient data.

**
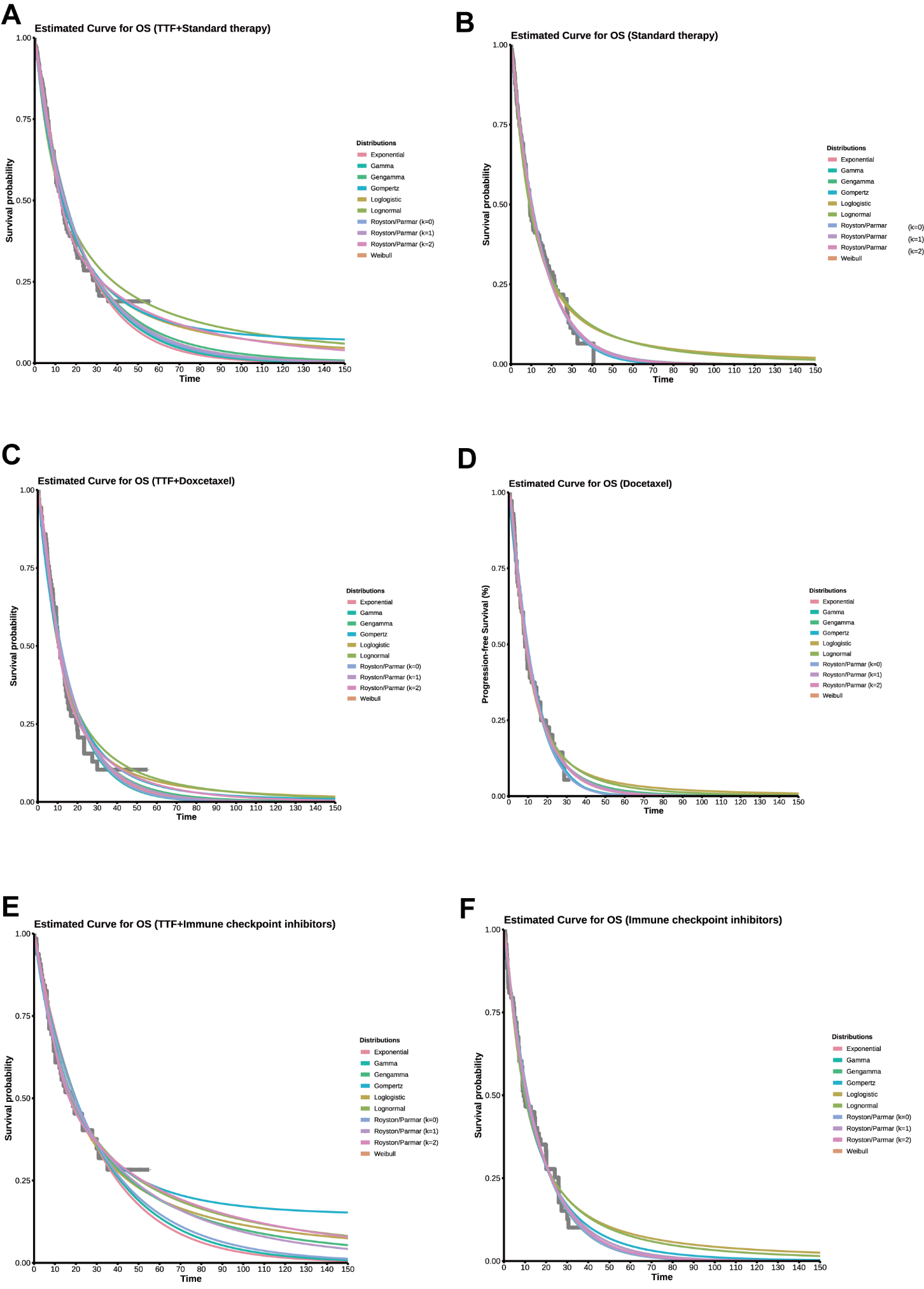
**

**
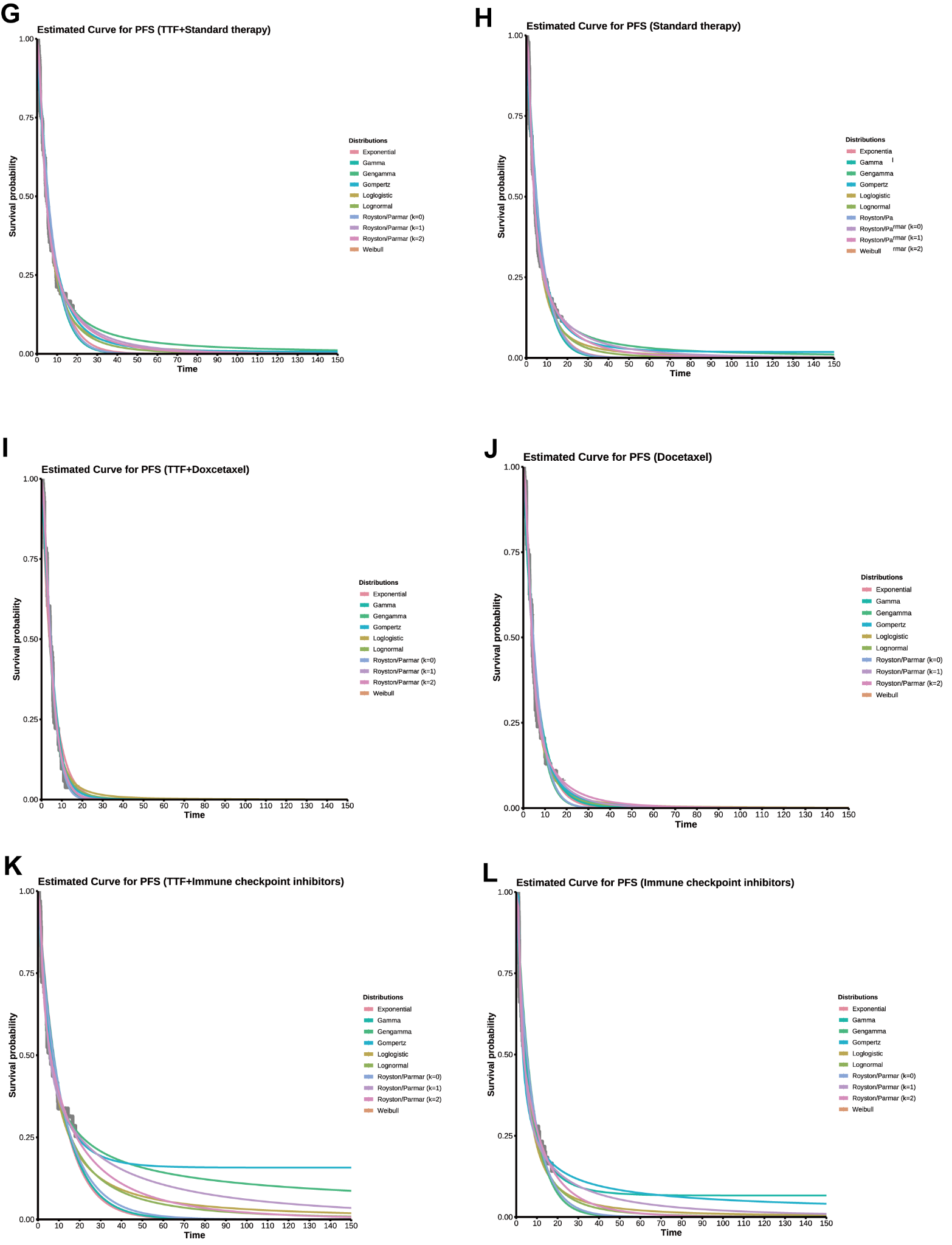
**

**
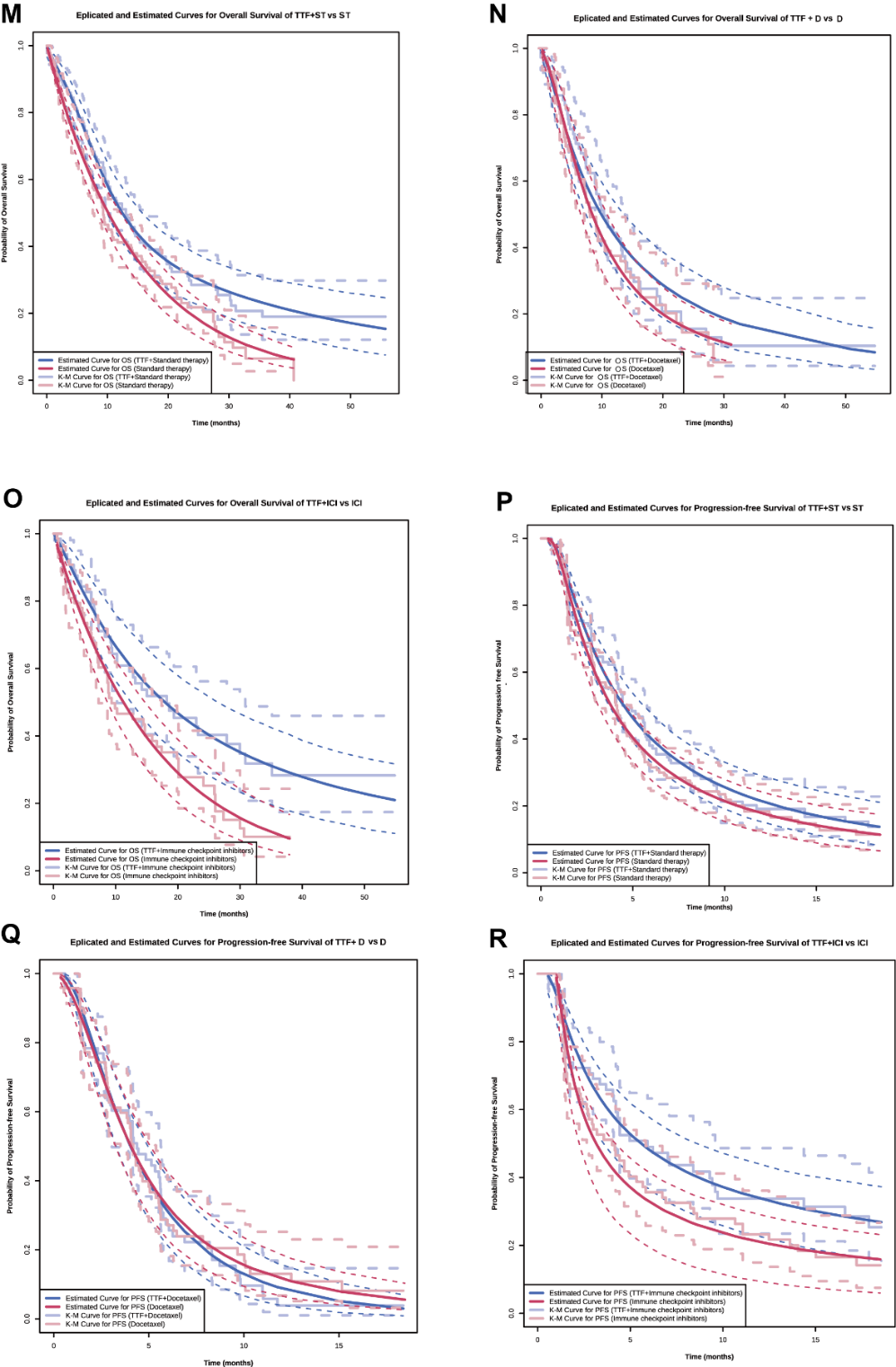
**

**Figure S2.** Estimated curves for (A-F) overall survival of all groups. Estimated curves for (G-L) progression-free survival of all groups. (M) Royston/Parmar and exponential distribution were used to estimate overall survival of the TTF + ST gourp and the ST group, respectively. (N) Loglogistic and lognormal distribution were used to estimate overall survival of the TTF + Docetaxel group and the Docetaxel group, respectively. (O) Loglogistic and exponential distribution were used to estimate overall survival of the TTF + ICI group and the ICI group, respectively. (P) Generalized gamma distribution were used to estimate progression-free survival of the TTF + ST gourp and the ST group. (Q) Lognormal and loglogistic distribution were used to estimate progression-free survival of the TTF + Docetaxel group and the Docetaxel group, respectively. (R) Generalized gamma distribution were used to estimate progression-free survival of the TTF + ICI group and the ICI group.***Abbr.*** *TTF = tumor treating fields; ST = standard therapy; ICI = immune checkpoint inhibitor; D = docetaxel; OS = overall survival; PFS = progression-free survival.*


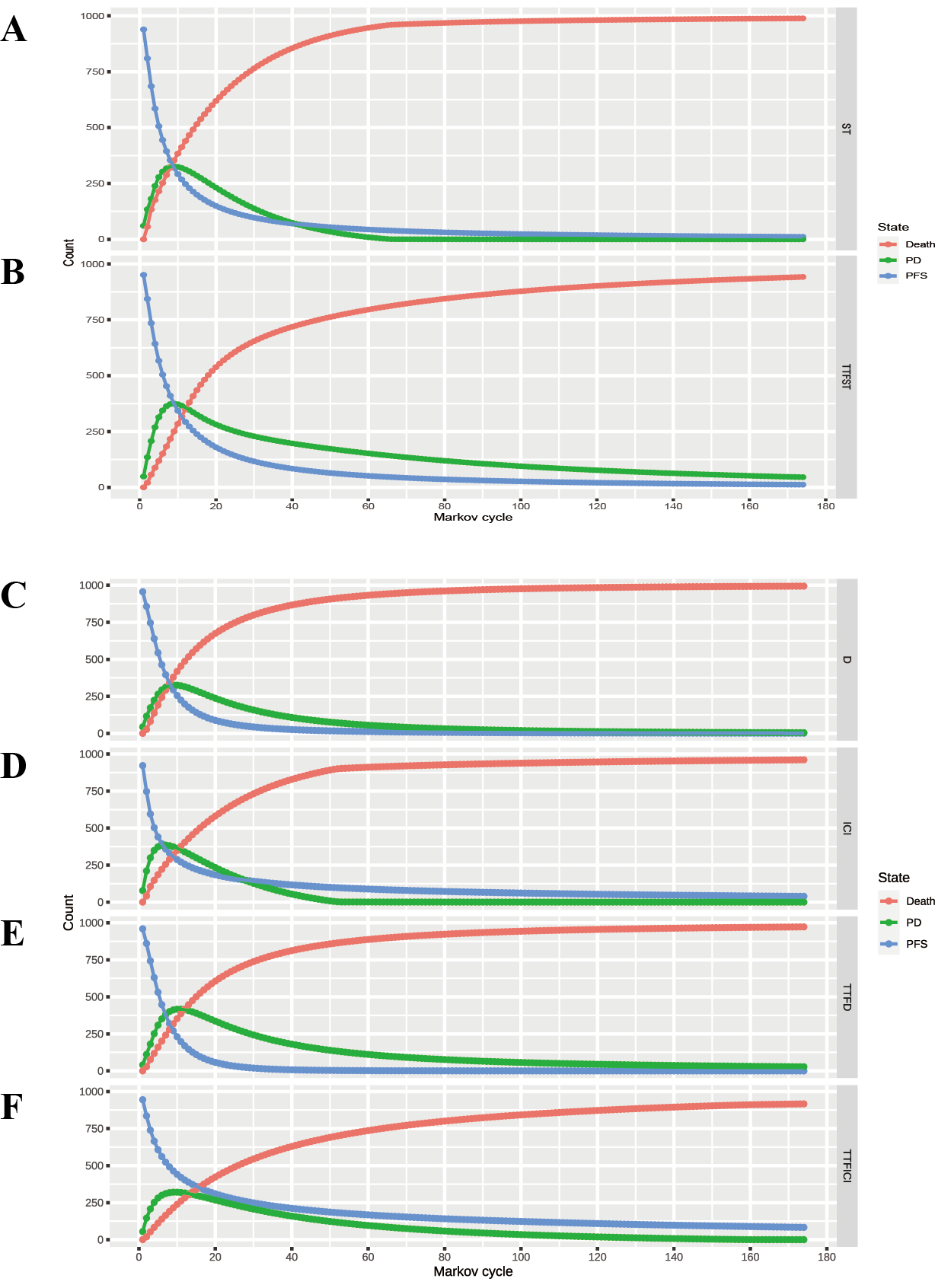


**Figure S3.** Patient counts of (A) Standard therapy group (B) TTF + Standard therapy group (C) Docetaxel group (D) Immune checkpoint inhibitors group (E) TTF + Docetaxel group (F) TTF + Immune checkpoint inhibitors group in 21-day Markov cycles for 10 years (174 cycles).


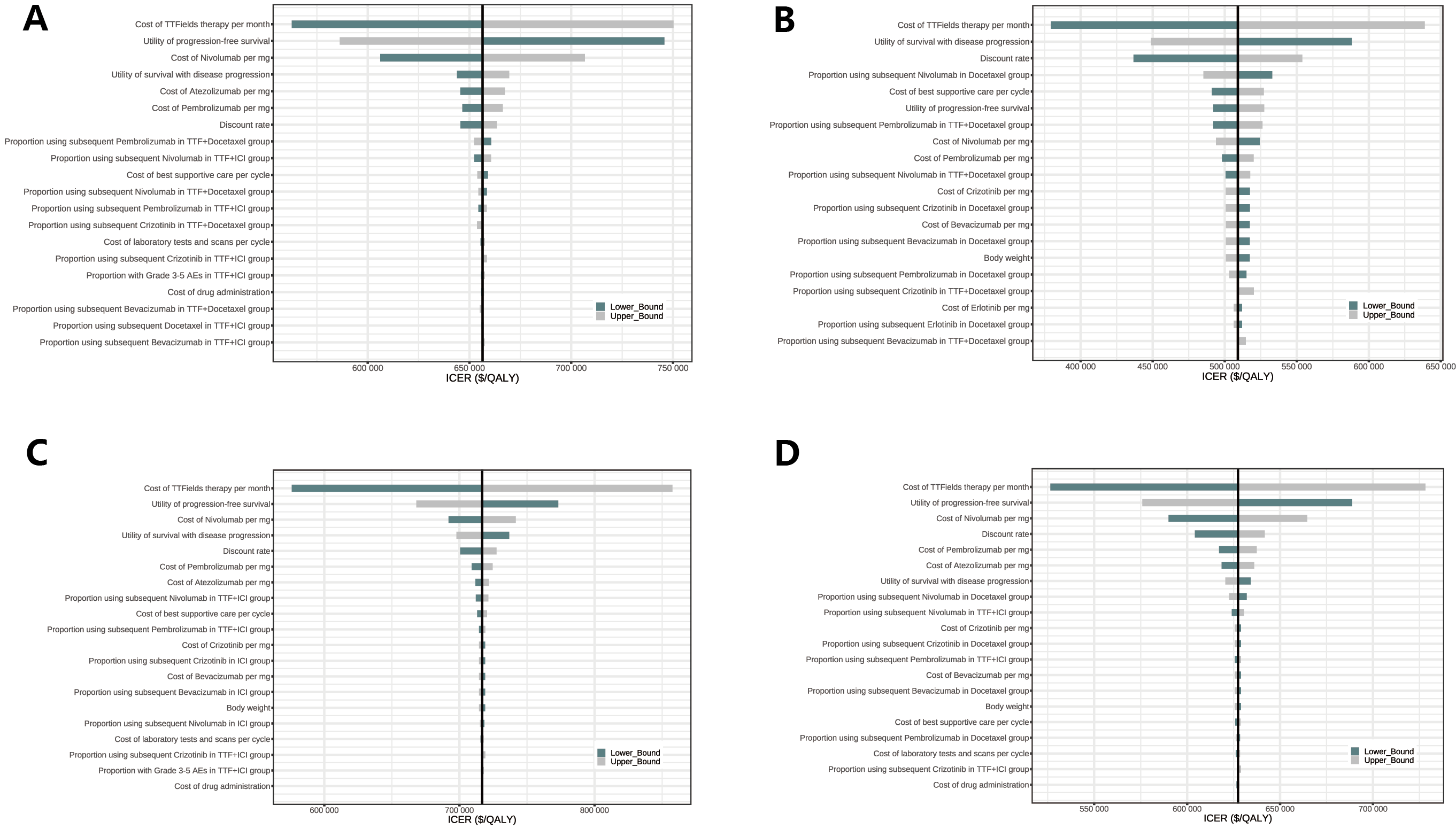


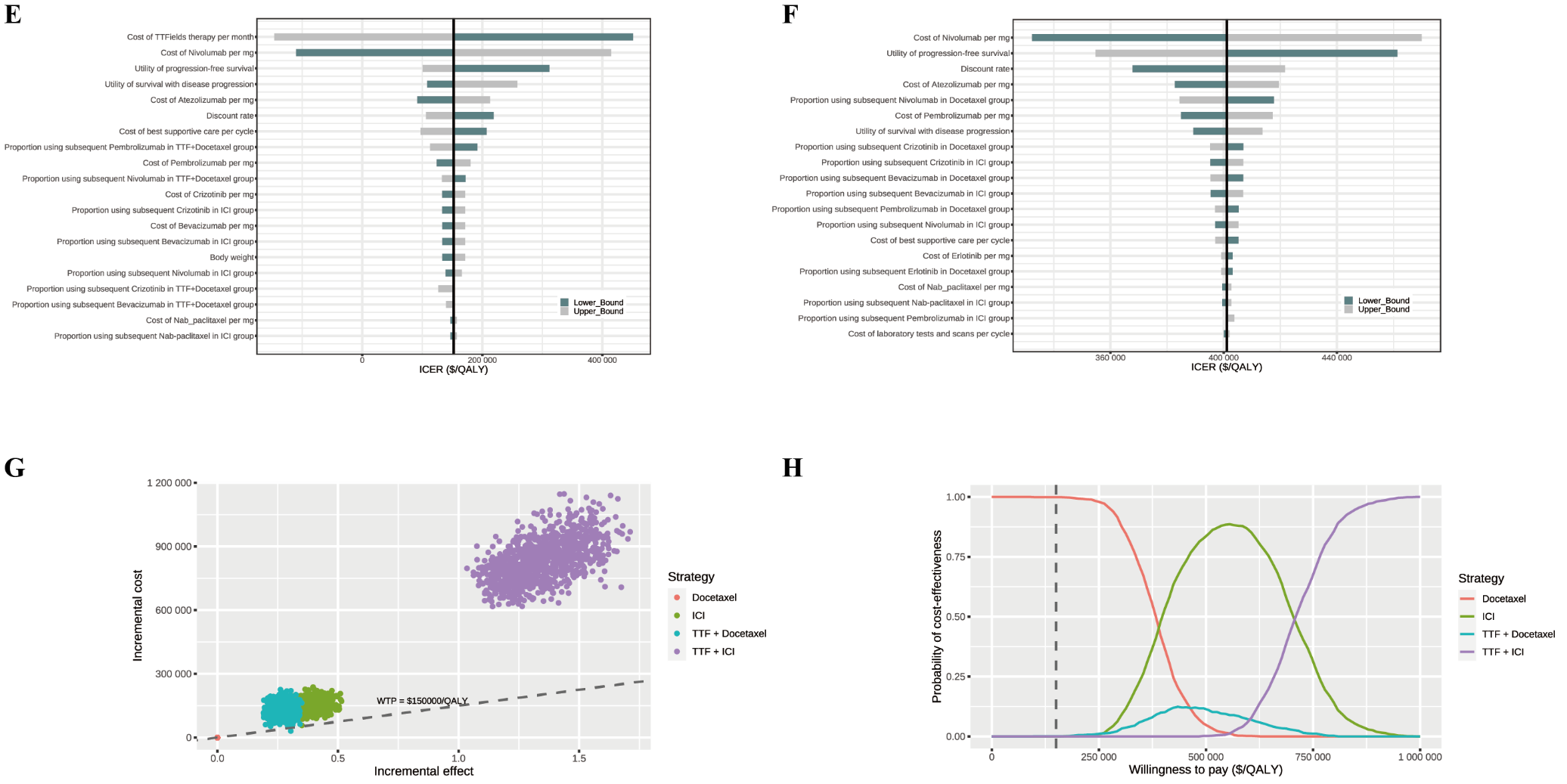


**Figure S4.** Sensitivity analyses of subgroup analysis. One-way sensitivity analysis for A) TTF + Immune checkpoint inhibitors vs TTF + Docetaxel; B) TTF + Docetaxel vs Docetaxel; C) TTF + ICI vs ICI; D) TTF + ICI vs Docetaxel; E) TTF + Docetaxel vs ICI; F) ICI vs Docetaxel; G) Incremental cost ($) and incremental effect (QALY) incurred by 1,000 probabilistic resamplings per strategy in the probabilistic sensitivity analysis; H) Probability of cost-effectiveness at varying willingness-to-pay. The dashed line represents the willing-to-pay threshold of $150,000 per QALY gained. ***Abbr.*** *TTF = tumor treating fields; ICI = immune checkpoint inhibitors; WTP = willingness-to-pay; QALY = quality-adjusted life-year.*


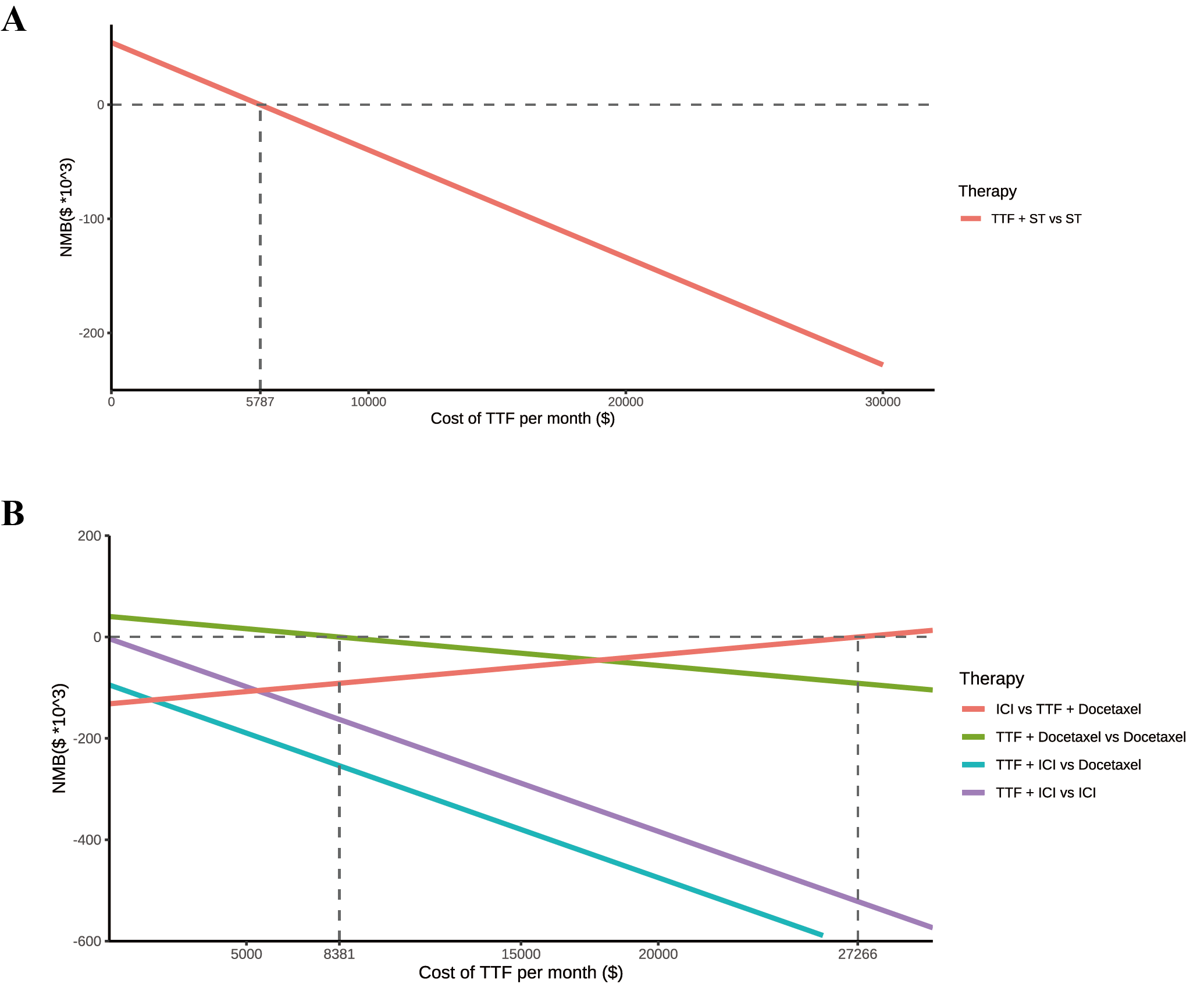


**Figure S5.** Impact of cost of TTF per month ($) on the INMB of comparisons between strategies in A) the main analysis and B) subgroup analysis. ***Abbr.*** *INMB = incremental net monetary benefit; TTF = tumor treating fields; ST = standard therapy; ICI = immune checkpoint inhibitor.*
